# Lactate as a Predictor of 30-Day Mortality in Cardiogenic Shock

**DOI:** 10.3390/jcm13071932

**Published:** 2024-03-27

**Authors:** Gregor Klemm, Sebastian Markart, Alexander Hermann, Thomas Staudinger, Christian Hengstenberg, Gottfried Heinz, Robert Zilberszac

**Affiliations:** 1 Department of Cardiology, Medical University of Vienna, 1090 Vienna, Austria; gregor@klemm.me (G.K.); christian.hengstenberg@meduniwien.ac.at (C.H.);; 2 Department of Internal Medicine I, Medical University of Vienna, 1090 Vienna, Austria

**Keywords:** cardiogenic shock, lactate, critical care

## Abstract

**Background/Objectives:** This study sought to evaluate the efficacy of various lactate measurements within the first 24 h post-intensive care unit (ICU) admission for predicting 30-day mortality in cardiogenic shock patients. It compared initial lactate levels, 24 h levels, peak levels, and 24 h clearance, alongside the Simplified Acute Physiology Score 3 (SAPS3) score, to enhance early treatment decision-making. **Methods:** A retrospective analysis of 64 patients assessed the prognostic performance of lactate levels and SAPS3 scores using logistic regression and AUROC calculations. **Results:** Of the baseline parameters, only the SAPS3 score predicted survival independently. The lactate level after 24 h (LL) was the most accurate predictor of mortality, outperforming initial levels, peak levels, and 24 h-clearance, and showing a significant AUROC. LL greater than 3.1 mmol/L accurately predicted mortality with high specificity and moderate sensitivity. **Conclusions:** Among lactate measurements for predicting 30-day mortality in cardiogenic shock, the 24 h lactate level was the most effective one, suggesting its superiority for early prognostication over initial or peak levels and lactate clearance.

## 1. Introduction

Cardiogenic shock (CS) may arise from various causes, including acute myocardial infarction (AMI) and decompensated heart failure. It constitutes a life-threatening condition of severe end-organ hypoperfusion due to impaired cardiac output caused by primary cardiac dysfunction, resulting in a persistent systolic blood pressure (SBP) below 90 mm Hg for at least 30 min, refractory to fluid resuscitation, or requiring the use of vasoactive agents to maintain the desired SBP target [1].

The role of lactate in intensive care and cardiogenic shock is increasingly emphasized due to its diagnostic and prognostic significance. Elevated lactate levels in these settings are a result of Type A or Type B lactic acidosis [2].

Type A lactic acidosis, common in cardiogenic shock, occurs due to tissue hypoxia. Inadequate oxygen supply leads to anaerobic metabolism, causing lactate accumulation. This type of lactic acidosis is often seen in severe cases of cardiogenic shock, where cardiac output is insufficient to meet the body’s oxygen demand, resulting in increased anaerobic glycolysis [3,4].

Type B lactic acidosis, in contrast, occurs without overt hypoxia. It can result from various causes, including liver dysfunction, which impairs lactate clearance, and the use of certain medications that interfere with lactate metabolism. Additionally, underlying metabolic disorders can contribute to Type B lactic acidosis by altering lactate production or clearance mechanisms [5].

The value of monitoring lactate in cardiogenic shock is well established. Lactate clearance, the rate at which lactate levels decrease, is closely linked to patient outcomes. Effective resuscitation in cardiogenic shock often leads to a rapid decrease in lactate levels, associated with improved survival rates. Conversely, inadequate lactate clearance is indicative of ongoing tissue hypoperfusion and is correlated with poorer outcomes [3,6].

Although the association between high lactate concentrations or low lactate clearance and mortality in cardiogenic shock has been repeatedly demonstrated, concrete statements and established, substantiated cut-off values for clinical practice are still lacking. Consequently, the predictive accuracy for individual cases remains limited. The most frequently investigated lactate parameters in cardiogenic shock include lactate concentration at admission and at specific time intervals (e.g., 8, 12, and 24 h) as well as lactate clearance over various periods. However, it remains unresolved which of these metrics is the most valid predictor of outcomes.

The aim of the present study was thus to examine various lactate parameters within the first 24 h for their predictive properties in a retrospective cohort of CS patients.

## 2. Materials and Methods

This is a monocentric retrospective data analysis.

### 2.1. Study Population

The study aimed to include data from all patients between 2017 and 2020 (estimated *n* = 2000) from two selected intensive care units at the University Hospitals for Internal Medicine I and II in the General Hospital Vienna:

- The internal medicine intensive care unit 13i2;
- The cardiology intensive care unit 13h3.

For this analysis, 600 out of the 2000 patients were screened. The data were retrospectively collected and subsequently analyzed.

Inclusion criteria specified that only patients admitted and treated in the specified time frame (01/2017 to 12/2020) at these intensive care units and who met the criteria for cardiogenic shock were included. Cardiogenic shock was defined as follows:

- SBP < 90 mmHg for >30 min or use of catecholamines to maintain a SBP > 90 mmHg;
- Clinical or radiological signs of pulmonary congestion;
- Reduced end-organ perfusion (neurological impairment, cold extremities, oliguria with <30 mL/h, or serum lactate concentration >2 mmol/L).

Excluded were patients under 18 years of age, pregnant patients, those treated for suicidal/autoaggressive actions, patients treated for intoxication, and patients missing at least one lactate parameter.

In this analysis, 600 patients were screened for cardiogenic shock. After considering the inclusion and exclusion criteria, 64 patients were included in the analysis. The study protocol complies with the Declaration of Helsinki and was approved by the ethics committee of the Medical University of Vienna; it was exempted from informed consent requirements owing to its retrospective observational study design.

### 2.2. Clinical Data

Data for the study were sourced from the Vienna General Hospital’s systems. Key sources were patient letters, surgical reports, radiological and laboratory results, and medical records. Anonymized data were transferred to an Excel spreadsheet and saved on an external USB drive, with personal identifiers replaced by pseudonyms or case numbers.

The retrospective analysis included demographic details (gender, age, height, and weight), ICU admission and discharge dates, ICU stay length, transfers, re-admissions, surgeries, deaths, diagnoses, pre-existing conditions (e.g., hypertension or COPD), shock at admission, prior surgeries, CPR events (cardiopulmonary resuscitation), SAPS-3 score, vital signs, echocardiographic findings, and ICU parameters like immunosuppression, blood gas, and electrolyte analysis.

This retrospective study involved no patient interventions, relying on standard ICU clinical routines and documentation for data. 

All data were anonymized post collection, and are not linkable to individuals except via a separate key file for specific cases. Data security was emphasized with password protection and access restricted to select personnel within the hospital network.

### 2.3. Lactate Measurements

Various lactate parameters within the first 24 h were studied for their predictive properties:

- The lactate concentration measured immediately after ICU admission (First Lactate; FL);
- The lactate concentration measured 24 h after ICU admission (Last Lactate; LL);
- The maximum lactate concentration measured in the first 24 h after ICU admission (Peak Lactate; PL);
- Lactate clearance is calculated based on the difference in lactate levels measured at two time points within the first 24 h of ICU admission, indicating the rate at which lactate is removed from the bloodstream in the first 24 h (LC). The formula for LC is as follows:

Lactate Clearance (=LC;%) = ((Initial Lactate Level (=FL; mmol/L) − Lactate Level after 24 h (=LL; mmol/L))/Initial Lactate Level (=FL; mmol/L)) × 100

LC is expressed as a percentage, with higher values indicating a more effective clearance of lactate from the bloodstream.

### 2.4. Statistical Analysis

Variables are expressed as median and quartiles (due to clearly non-normal distributions for most variables) for continuous parameters, and counts and percentages for categorical variables.

All demographic data and patient characteristics collected were subjected to descriptive analysis. Qualitative attributes were depicted graphically as bar diagrams and described in terms of frequency percentages. Quantitative features were primarily described through their mean values, standard deviations, and range (minimum and maximum values), and were visually represented using bar graphs, box plots, or histograms.

Simple logistic regression analyses were conducted to investigate the relationship between various baseline variables and mortality. These analyses provided odds ratios (OR) and their 95% confidence intervals to quantify associations. A *p*-value of less than 0.05 was defined as denoting statistical significance.

Our primary objective was to assess key baseline characteristics, particularly focusing on different lactate variables, including initial lactate levels, levels after 24 h, the highest level within the first 24 h, and 24 h clearance, for their correlation with 30-day mortality. We employed logistic regression analyses to evaluate the predictive value of these lactate variables, followed by a comparison of their effectiveness. Further, Receiver Operating Characteristic (ROC) curve analysis was utilized to compare the Area Under the Curve (AUC) values of these variables. We also presented cut-off values for the four lactate variables, detailing their sensitivity, specificity, positive predictive value, and negative predictive value.

The exploratory hypotheses were tested at a significance level α of 0.05. Those lactate variables that demonstrated significant results in ROC analysis were subsequently subjected to the DeLong test to identify significant differences. The significance level for this test was adjusted using the Bonferroni correction to α = 0.05/3 ≈ 0.01667.

For statistical computations, the software SPSS^®^ (IBM, Armonk, NY, USA, V. 29.0) and MedCalc^®^ (MedCalc Software Ltd., Ostend, Belgium, V. 20.218) were utilized.

## 3. Results

### 3.1. Patient Demographics, Admission Details, and Intensive Care Unit Mortality Statistics 

This analysis encompassed 64 patients presenting with cardiogenic shock. Detailed baseline characteristics are delineated in Table 1. Of these patients, 21 (32.8%) were admitted postoperatively, while 25 (39.1%) were admitted subsequent to cardiopulmonary resuscitation. The remaining 18 patients developed cardiogenic shock following primary myocardial dysfunction like acute myocardial infarction (*n* = 14), severe valvular heart disease (*n* = 3), or refractory tachycardic atrial fibrillation (*n* = 1). Seven out of all patients were transferred from external medical facilities. The median duration of stay in the ICU was 9 days (quartiles 4–22). Overall, 23 of the 64 patients (35.9%) succumbed within 30 days of ICU admission, compared with 41 (64.1%) who survived. Thus, the total 30-day mortality rate within this cohort was 35.9%.

### 3.2. Postoperative Patient Analysis and Surgical Procedure Overview 

**Figure 1:**
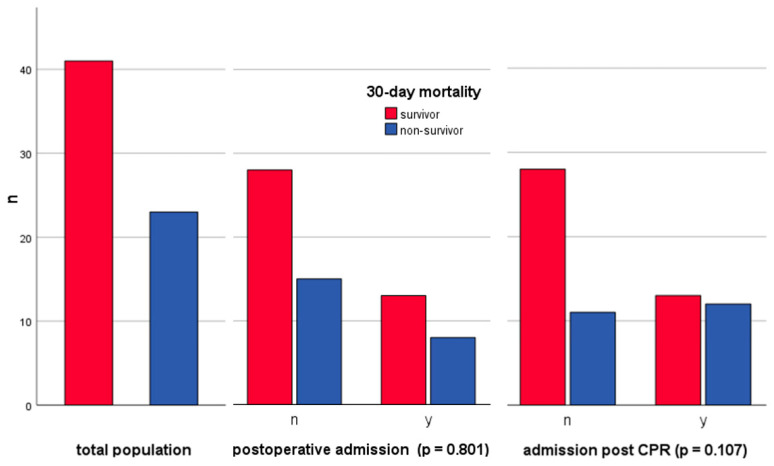
Numbers of 30-day mortality in total and according to admission reason: postoperative vs. nonsurgical patients and post-resuscitation vs. non-post-resuscitation patients.

Among the study cohort, 21 patients (32.8%) required ICU admission following surgical interventions. In this particular subgroup, the 30-day mortality rate was 38.1%, which did not significantly differ from the mortality rate found in the group of patients who had not undergone surgery (34.9%, with a *p*-value of 0.801). Additionally, the mortality rate within this subgroup was comparable to the 30-day mortality rate of 35.9% observed across the entire cohort, as shown in Figure 1. The surgeries predominantly consisted of cardiothoracic and vascular procedures (20 out of 21), encompassing 9 Coronary Artery Bypass Grafting (CABG) surgeries (4 of which included valve replacement), 7 diverse open cardiac surgeries (including Bentall procedures, pulmonary homograft, aortic valve replacement, mitral valve replacement, and valve reconstructions), 3 medical device implantations (e.g., pacemaker, Right or Left Ventricular Assistance Device (RVAD/LVAD)) and one interventional procedure (transfemoral aortic valve replacement, TAVI). A single patient was admitted post major abdominal surgery.

### 3.3. Cardiopulmonary Resuscitation: Admissions, Mortality, Rhythms, and Outcome Correlations 

Within the patient cohort, 25 out of 64 individuals were admitted during or immediately following CPR. The 30-day mortality in this subset was 52% (12 out of 25), and therefore almost 20 percentage points higher than in the remaining cohort (11 out of 39, 28%, *p* = 0.107) and about 12 percentage points above the aggregate cohort mortality rate (35.9%). The initial cardiac rhythm at the onset of resuscitation efforts was documented, with 52% (13 out of 25) exhibiting a shockable rhythm (Table 2). Predominantly, these rhythms were identified as ventricular fibrillation (VF; 11 out of 13). In two cases, the rhythm was classified as shockable, albeit undetermined which rhythm exactly. Of the initial non-shockable rhythms (*n* = 10), 7 were categorized as pulseless electrical activity (PEA) and 3 as asystole. Post resuscitation, initial rhythm remained undetermined in two cases. The median CPR duration was 30 (18–56) minutes across the whole cohort and did not differ significantly between non-survivors and survivors, with durations of 43 (30–60) minutes and 22 (12–40) minutes (*p* = 0.085). The shortest and longest CPR durations were 15 and 120 min for non-survivors, and 4 and 120 min for survivors, respectively. 

### 3.4. Logistic Regression Analyses: Baseline Characteristics

In the simple logistic regression analyses, only the SAPS3 score at admission (*p* = 0.022, OR = 1.044) demonstrated a significant association with 30-day mortality (Table 3). No significant relationships were found for the other variables tested, including sex, age, Body Mass Index (BMI), duration of ICU stay, CPR prior to admission, and number of total pre-existing conditions.

### 3.5. Graphical and Descriptive Analysis of Lactate Variables

Details regarding lactate concentrations are given in Table 4. 

The median concentration of FL was 3.5 (2.2–5.7) mmol/L. Among the deceased, this value was 4.0 (2.2–7.0), and among the survivors, it was 3.2 (2.0–5.2; *p* = 0.378). The maximum FL values varied strongly (18 vs. 11.7 mmol/L), while the minimum values were similar (1.1 vs. 0.9 mmol/L). 

The median value of LL was 2.0 (1.2–3.3) mmol/L across the entire population. For the deceased, this value (3.4, 1.8–5.4) was significantly (*p* = 0.001) higher compared to the survivors (1.7, 1.1–2.4). The maximum LL values were 12.8 and 13.2 mmol/L, while the minimum LL value was the same at 0.7 mmol/L.

The highest PL was also significantly higher among the patients who died within 30 days (5.6, 3.1–10.1 mmol/L vs. 3.7, 2.3–6.9 mmol/L; *p* = 0.040). The maximum values were 21 and 16 mmol/L, and the minimum values were 1.7 and 0.9 mmol/L, respectively. 

The median LC was 36.0 (7.3–57.1)% across the entire population. This varied significantly between the deceased and survivors: 15.0 (−35.0–48.3)% and 44.2 (27.3–63.6)% (*p* = 0.023). While the minimum LC values were markedly different (−364.0 vs. −169.4%), the maximum LC values were similar (84.1 vs. 88.1%). 

The SAPS3 score (*n* = 55) was also included as it is an established prognostic marker [7] and serves as a point of comparison. The SAPS3 score was not obtainable in nine cases, seven among survivors (17.1%) and two among non-survivors (8.7%). The median SAPS3 score at admission was 70 (57–84) points. In the group of deceased patients, the mean was 82 (61–90), with a maximum of 125 points and a minimum of 48. For survivors, all three values were lower: median 67 (53–79), maximum 95, and minimum 40. Therefore the SAPS3 score differed significantly between these two groups (*p* = 0.025)

**Figure 2:**
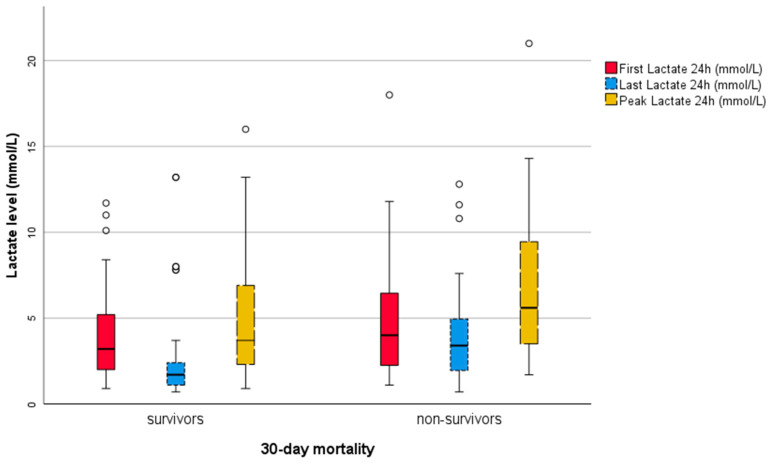
Boxplots of FL, LL, and PL by 30-day mortality. black circles indicating outliers.

**Figure 3:**
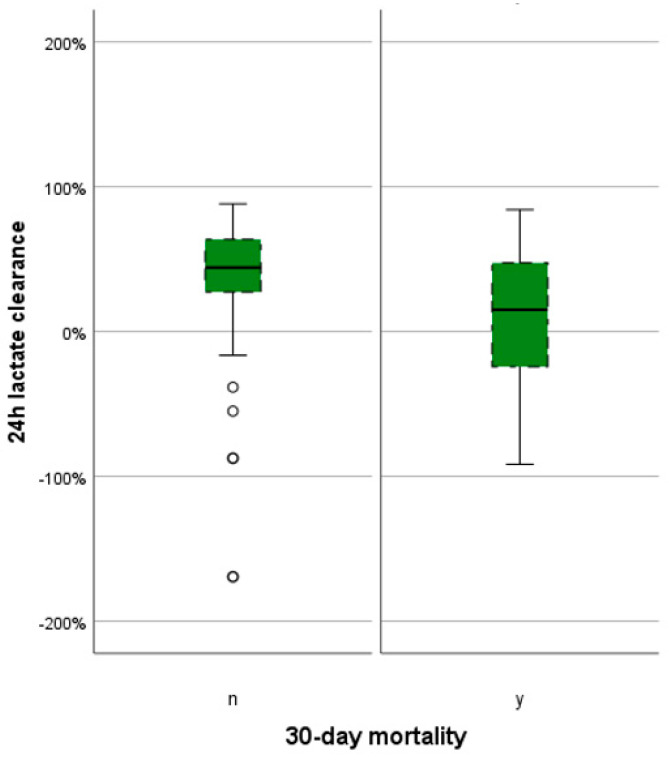
Boxplot of LC by 30-day mortality. black circle indicating outliners.

The five described prognostic variables (FL, LL, PL, LC, and SAPS3) are graphically represented in box plots, comparing 30-day mortalities, in Figure 2 and Figure 3.

### 3.6. Scatter Diagrams and Correlations

**Figure 4:**
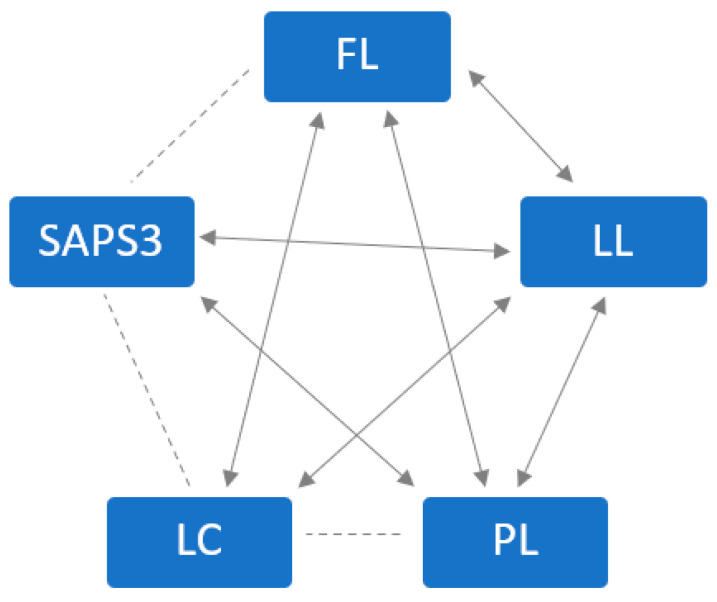
Correlations in between the five prognostic markers.

To demonstrate correlations among various prognostic markers, a correlation analysis was conducted using Spearman’s correlation coefficient, and the results were tested for statistical significance (*p* < 0.05). These correlations are also graphically represented in Figure 4. LL showed a significant correlation with all four other variables studied (see Table 5: Correlations of lactate variables and SAPS3). PL exhibited a significant correlation with three other variables: LL, FL, and SAPS3, but not with LC. FL also displayed a significant correlation with all variables except SAPS3. LC significantly correlated with FL and LL, but not with SAPS3 and PL. SAPS3, in turn, showed significant correlation with two other variables: LL and PL.

### 3.7. Simple Logistic Regression Analyses 

The association of 30-day mortality with four lactate parameters (LL, PL, LC, and FL) and SAPS3 was evaluated. Significant associations with 30-day mortality were only found for LL (*p* = 0.020, OR 1.289). No significant association could be shown for FL (*p* = 0.197), PL (*p* = 0.067), and LC (*p* = 0.053). Details are shown in Table 6. 

### 3.8. Multivariate Logistic Regression Analyses 

Significant lactate variables (LL) from the simple logistic regression analysis were subsequently used in multivariate logistic regression analyses with baseline variables that also showed significant associations with ICU mortality in the simple analysis (SAPS3).

LL did not show significant results in the multivariant logistic regression analysis with SAPS3 as covariant (*p* = 0.059). Also, SAPS3 did not show a *p* < 0.05 in this model (*p* = 0.070). Details are shown in Table 7.

### 3.9. ROC Analysis 

**Figure 5:**
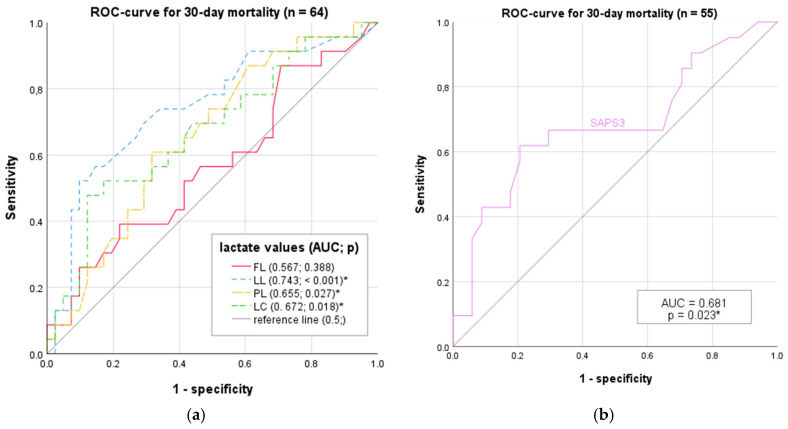
Diagnostic accuracy of lactate measurements and SAPS3 score in mortality prediction. (**a**) The blue curve indicates Last Lactate within 24 h (LL), the red curve indicates First Lactate (FL), the green curve indicates Lactate Clearance within first 24 h (LC), and the yellow curve indicates Peak Lactate within 24 h (PL). (**b**) The purple curve in (**b**) represents SAPS3 score. The diagonal grey line represents the line of no discrimination (chance prediction) in both figures. Each line in this ROC graph represents the sensitivity versus 1-specificity of the respective values taken in the first 24 h of ICU admission, predicting 30-day mortality. * marking significant results in the ROC-analysis regarding 30-day mortality.

To quantify and compare the diagnostic accuracy of individual lactate values with respect to ICU mortality, a Receiver Operating Characteristic (ROC) curve analysis was conducted (see Figure 5a,b). The diagnostic properties, sensitivity, and specificity (or 1-specificity) are presented. The area under these ROC curves (Area Under Curve, AUC or AUROC) was exploratively determined for the mentioned variables.

The largest AUC in the ROC analysis was observed for LL (AUC 0.743, *p* < 0.001), followed by SAPS3 (AUC 0.681, *p* = 0.023) and LC (AUC 672, *p* = 0.018). PL displayed an AUC of 0.655, which was still significant (*p* = 0.027). Only FL showed a non-significant result with an AUC of 0567 (*p* = 0.388). These are also listed in Table S1 (Supplementary Materials).

The values FL, LL, PL, and LC were available for every included patient (*n* = 64). In the ROC analysis of SAPS3, *n* is 55, due to nine missing data points.

### 3.10. Diagnostic Characteristics 

In the ROC analysis, the Youden Index (YI) was calculated for five variables as an example of a cut-off value. These cut-offs are detailed in Table S1 with diagnostic quality measures including sensitivity, specificity, positive predictive value (PPV), and negative predictive value (NPV).

No significant AUC was found for FL in the ROC analysis (0.567, *p* = 0.388). A YI of 0.172 at >5.5 mmol/L yielded a sensitivity of 39.1% and a specificity of 78.1%. Once FL reached >6.7 mmol/L, the specificity rose to 90.2%, but sensitivity remained low at 26.1%.

The YI for LL (0.424) was established at >3.1 mmol/L, affecting 16 patients, with a sensitivity of 52.2% and a specificity of 90.2%. Both PPV and NPV exceeded 75%. At a cut-off of >2.4 mmol/L, sensitivity increased to 60.9%, with a corresponding specificity decrease to 78.1%. A cut-off of >5 mmol/L resulted in a specificity of 92.7% but a sensitivity of only 26.1%.

When the PL within the first 24 h exceeded the normal range (>2 mmol/L), sensitivity was 95.7%, but specificity was only 19.5%. The YI for PL was found to be 0.292 at >5 mmol/L, with a sensitivity of 60.9% and a specificity of 68.3%. A PL > 10 mmol/L indicated a sensitivity of 26.1% and a specificity of 87.8%. There were eleven instances of PL > 10 mmol/L, two >15, and one >20 mmol/L.

The YI for LC was identified at <10% (0.356), with a sensitivity of 47.8% and a specificity of 87.8%. A negative LC (<0; *n* = 14) corresponded to a specificity of 87.8% and a sensitivity of 39.1%.

For SAPS3, the YI of 0.413 at >79 points revealed a sensitivity of 61.9% and a specificity of 79.4%. At a threshold of >85 points (*n* = 12), the specificity exceeded 90% with a sensitivity of 42.9%.

### 3.11. DeLong Test for AUC Differences 

The lactate variables that demonstrated significant results in explorative simple logistic regression analyses and ROC analyses (*p* < 0.05) were subsequently tested. The DeLong test was used to compare the AUCs of LL, LC, and PL for significant differences, with the significance level adjusted according to Bonferroni (*p* < 0.0167).

The DeLong test revealed no significant differences between the AUCs of LC and LL (ΔAUC 0.071, *p* = 0.333), between LC and PL (ΔAUC 0.016, *p* = 0.877), or between LL and PL (ΔAUC 0.088, 0.140) (Table S2, Supplementary Materials). 

Additional variables (FL and SAPS3) were also included in an explorative analysis and tested against each other using the DeLong test (see Table S3, Supplementary Materials). A significant difference was only found when comparing the AUCs of LL and FL (0.176, *p* = 0.019). No significant results were observed in the other AUC comparisons.

## 4. Discussion

Our study examined 64 cardiogenic shock patients, revealing baseline characteristics in line with recent research. With a median age of 64, predominantly male patients (72%), and a median BMI of 26.2, our cohort is similar to those in studies by Fuernau et al. [8] (69 years, 71% male, BMI 27, median lactate at admission 3.9 mmol/L) and Lindholm et al. [4] (67 years, 75% male, median baseline lactate 2.8 mmol/L). 

Variations in inclusion and exclusion criteria among studies impact comparability. For instance, 39% of our patients were admitted post resuscitation and 34% post operation, differing from Lindholm et al. [4], who excluded postoperative admissions, and Fuernau et al. [8], who omitted patients with extended cardiopulmonary resuscitation. Park et al. [6] excluded prehospital resuscitations. Hussain et al.’s cohort [9] included 22% perioperative patients.

In our investigation, the SAPS3 score was identified as a univariate predictor of 30-day mortality (*p* = 0.022, OR 1.044), echoing the findings of previous studies [7,10]. However, its effectiveness was not confirmed in multivariate analysis, indicating possible limitations for individual prognostication. This observation aligns with the understanding that the SAPS3 score, fundamentally a logistic model, may not encompass the dynamic intricacies of patient conditions. Furthermore, lactate, not initially incorporated into the SAPS3 model, has been recognized for its potential to improve the model’s prognostic accuracy [11]. The absence of lactate highlights a gap in the SAPS3 model, pointing to the benefit of integrating simple, validated indicators to refine prognostication. 

We found no significant differences in 30-day survival rates among various demographics and clinical variables, including post-resuscitation status. This lack of significant difference, albeit with a trend towards higher mortality in post-resuscitation admissions (52%) compared to others (32%), aligns with the findings from recent studies that also report no significant disparity in survival outcomes between CPR recipients and non-recipients for out-of-hospital cardiac arrest (OHCA) [12,13,14,15]. It is of note that most recently, it has been demonstrated that in-hospital cardiac arrest (IHCA) patients tend to have poorer outcomes compared to OHCA patients [16], which suggests a nuanced understanding of CPR outcomes based on the setting of the cardiac arrest.

Acknowledging the methodological challenges highlighted previously [17], we recognize the complexity of jointly analyzing cardiogenic shock (CS) and resuscitated patients. The critique suggests potential differences in outcomes and predictors that could be masked when these groups are combined. However, given our study’s relatively small sample size, a separate analysis would not have been feasible or statistically robust. 

In basic statistical testing, we observed a higher prevalence of coronary artery disease among patients who succumbed; nonetheless, this relationship did not reach statistical significance in a simple logistic regression analysis that factored in total baseline comorbidities. It is worth noting that biochemical markers of cardiac ischemia have previously been documented to independently correlate with mortality among ICU patients [18].

Our 30-day mortality rate was 35.9%, aligning with Torgersen et al.’s [19] 28-day mortality of 30% and Fuernau et al.’s [8] 30-day mortality of 38%. 

Contrasting with Valente et al. [20] and Lindholm et al. [4], who identified baseline lactate as a strong mortality predictor, our median FL of 3.5 mmol/L was not significantly different between survivors and non-survivors. PL as well as LC were higher in non-survivors, although not significant different in univariate logistic regression analysis. LL was the most predictive variable for 30-day mortality, in agreement with Ferreruela et al. [21] and Fuernau et al. [8]. The Youden Index for LL at >3.1 mmol/L demonstrated high specificity (90.2%) and moderate sensitivity (52.2%). Our study thus emphasizes the importance of early shock reversal in cardiogenic shock, a concept strongly supported by previous studies [22,23,24]. This includes pharmacological and possibly mechanical support, as well as rigorous hemodynamic monitoring to improve tissue perfusion, as indicated by decreasing lactate levels [25].

The study’s observation that FL and PL have less prognostic significance suggests that initial lactate levels at ICU admission may not fully represent a patient’s recovery potential. In this context, Levy et al. [26] emphasize the dynamic nature of shock management, advising against prematurely discontinuing therapy based on initial severity indicators. Our study thus emphasizes the need for early, aggressive, and sustained intervention in cardiogenic shock. Clinicians are encouraged to adopt a persistent, adaptive treatment strategy, drawing parallels from early goal-directed therapy (EGDT) in sepsis management [27,28]. This involves the ongoing reassessment and adaptation of therapeutic strategies based on evolving clinical and biochemical markers.

Future analyses will explore larger cohorts to further investigate the relationship between LL and LC, and long-term outcomes post cardiogenic shock.

### Limitations and Strengths

This study has some limitations, including its small sample size, retrospective nature, and single-center scope. These aspects may influence the broader applicability of our findings. Limitations in documentation, such as inconsistent parameters and missing data, affect the robustness of our conclusions. The patient cohort’s size and diversity, along with pre-ICU care variations and potential biases in lactate measurement due to medical interventions, also impact our findings’ generalizability. Despite these constraints, this study’s strength lies in its meticulous documentation review and expert resolution of diagnostic ambiguities, ensuring the comprehensive and accurate inclusion of cardiogenic shock cases.

## 5. Conclusions

In terms of predicting 30-day mortality in cardiogenic shock, the lactate concentration after 24 h was superior to the concentration on admission, the peak concentration, and the 24 h clearance. At a value of >3.1 mmol/L, it had a specificity of 90.2% and a sensitivity of 52.2% for predicting the 30-day survival of patients with cardiogenic shock.

## Figures and Tables

**Table 1:** Baseline patient characteristics.

	Total (*n* = 64)	30-day Mortality		*p*-Value
		Non-Survivors (*n* = 23)	Survivors (*n* = 41)	
Baseline characteristics				
Gender (female)	18 (28%)	8 (35%)	10 (24%)	0.375 ^1^
Age (years)	64 (55–72)	66.5 (60–76)	62 (46–70)	0.071 ^2^
BMI (kg/m^2^)	26.2 (23.9–30.6)	25.2 (23.9–30.9)	26.6 (23.9–30.6)	0.876 ^2^
ICU-stay				
SAPS3 Score at admission	70 (57–84)	82 (61–90)	67 (53–79)	0.025 ^2,^*
Admission post CPR	25 (39%)	12 (52%)	13 (32%)	0.107 ^1^
Admission post surgery	21 (33%)	8 (35%)	13 (32%)	0.801 ^1^
Duration of ICU stay (days)	9 (4–22)	10 (3–23)	9 (6–18)	0.445 ^2^
Risk factors				
Immunosuppression	2 (3%)	1 (4%)	1 (2%)	0.365 ^1^
Hypertension	29 (48%)	9 (39%)	20 (49%)	0.457 ^1^
Coronary artery disease	39 (61%)	18 (78%)	21 (51%)	0.033 ^1,^*
Peripheral artery disease	10 (16%)	4 (17%)	6 (15%)	0.771 ^1^
Diabetes mellitus	13 (20%)	3 (13%)	10 (24%)	0.279 ^1^
Chronic kidney disease	14 (22%)	5 (22%)	9 (22%)	0.984 ^1^
COPD	6 (9%)	3 (13%)	3 (7%)	0.451 ^1^
Malignant disease	3 (5%)	2 (9%)	1 (2%)	0.256 ^1^

*n* (%) for categorical and median (quartiles) for continuous variables. Comorbidities referring to diagnoses documented before admission to ICU. ^1^ Chi^2^–Test, ^2^ Mann–Whitney U Test, * significant to α = 0.05. Abbreviations: BMI = Body Mass Index; ICU = Intensive care unit; SAPS = Simplified Acute Physiology Score; CPR = Cardiopulmonary resuscitation; COPD = Chronic obstructive pulmonary disease.

**Table 2:** Admitted after CPR: Analysis of clinical characteristics.

	Total	30-Day Mortality	
		Non-Survivors	Survivors
CPR Pre-ICU	*n* = 25	*n* = 12	*n* = 13
First Rhythm CPR			
Total shockable	13 (52%)	4 (33%)	9 (69%)
Ventricular fibrillation, *n* (%)	11 (44%)	4 (33%)	7 (54%)
Unspecified, shockable, *n* (%)	2 (8%)	0 (0%)	2 (15%)
Total non-shockable, *n* (%)	10 (40%)	6 (50%)	4 (31%)
Asystole, *n* (%)	3 (12%)	1 (8%)	2 (15%)
Pulseless electrical activity, *n* (%)	7 (28%)	5 (42%)	2 (15%)
Unspecified	2 (8%)	2 (17%)	0 (0%)
CPR duration [min]			
Maximum CPR duration [min]	120	120	120
Minimum CPR duration [min]	4	15	4
Median CPR duration [min]	30 (18–56)	43 (30–60)	22 (12–40)

*n* (%) for categorical and median (quartiles) for continuous variables. Abbreviation: CPR = Cardiopulmonary resuscitation.

**Table 3:** Simple logistic regression analyses of baseline variables.

30-day Mortality	Regression Coefficient	*p*	Odds Ratio	95% CI	
Sex (male)	−0.503	0.377	0.605	0.198	1.845
Age [Years]	0.041	0.066	1.043	0.997	1.088
BMI [kg/m^2^]	−0.002	0.970	1.042	0.899	1.108
Days ICU stay	−0.006	0.731	0.994	0.963	1.027
SAPS 3 Score *	0.043	0.022 *	1.044	1.006	1.083
CPR prior to ICU	−0.854	0.111	0.426	0.149	1.216
Total comorbidities	0.096	0.588	1.101	0.777	1.560

* = significant association with 30-day mortality in simple logistic regression analysis; Abbreviations: CI = Confidence interval, ICU = Intensive care unit, SAPS = Simplified Acute Physiology Score.

**Table 4:** Descriptive analysis of lactate variables and SAPS.

Descriptive Analysis	Unit	Total	30-Day Mortality		*p*-Value *
			Non-Survivors	Survivors	
FL [mmol/L]	Median (quartiles)	3.5 (2.2–5.7)	4.0 (2.2–7.0)	3.2 (2.0–5.2)	0.378
	Maximum	18.0	18.0	11.7	-
	Minimum	0.9	1.1	0.9	-
LL [mmol/L]	Median (quartiles)	2.0 (1.2–3.3)	3.4 (1.8–5.4)	1.7 (1.1–2.4)	0.001
	Maximum	13.2	12.8	13.2	-
	Minimum	0.7	0.7	0.7	-
PL [mmol/L]	Median (quartiles)	4.3 (2.6–8.6)	5.6 (3.1–10.1)	3.7 (2.3–6.9)	0.040
	Maximum	21.0	21.0	16.0	-
	Minimum	0.9	1.7	0.9	-
LC [%]	Median (quartiles)	36.0 (7.3–57.1)	15.0 (−35.0–48.3)	44.2 (27.3–63.6)	0.023
	Maximum	88.1	84.1	88.1	-
	Minimum	−364.0	−364.0	−169.4	-
SAPS3	Median (quartiles)	70 (57−84)	82 (61–90)	67 (53–79)	0.025
	Maximum	125	125	95	-
	Minimum	40	48	40	-

Abbreviations: LL = Last Lactate; LC = Lactate Clearance; PL = Peak Lactate; FL = First Lactate; SAPS3 = SAPS 3-Score; * the Mann–Whitney U test was employed to assess group differences.

**Table 5:** Correlations between lactate variables and SAPS3.

Correlation	Spearman’s Correlation Coefficients (*p*-Value)				
	LL	FL	LC	PL	SAPS3
LL	1 (/)	0.481 (<0.001) *	−0.535 (<0.001) *	0.637 (<0.001) *	0.269 (0.047) *
FL	-	1 (/)	0.430 (<0.001) *	0.825 (<0.001) *	0.243 (0.074)
LC	-	-	1 (/)	0.063 (0.618)	−0.107 (0.435)
PL	-	-	-	1 (/)	0.269 (0.047) *
SAPS3	-	-	-	-	1 (/)

* significant correlation (*p* < 0.05). / no test was executed. Abbreviations: LL = Last Lactate; LC = Lactate Clearance; PL = Peak Lactate; FL = First Lactate; Spearman’s rank correlation coefficient *p* < 0.05.

**Table 6:** Simple logistic regression analysis: Lactate variables.

Simple Logistic Regression	*p*-Value	Regression Coefficient	Odds Ratio	Odds Ratio 95%-CI	
FL	0.222	0.100	1.106	0.941	1.299
LL	0.020 *	0.254	1.289	1.041	1.596
PL	0.067	0.122	1.129	0.992	1.286
LC	0.053	−0.010	0.990	0.980	1.000
SAPS3	0.022 *	0.043	1.044	1.006	1.083

* significant association with 30-day mortality in simple logistic regression analysis (*p* < 0.05). Abbreviations: LL = Last Lactate; LC = Lactate Clearance; PL = Peak Lactate; FL = First Lactate, SAPS3 = SAPS-3 score.

**Table 7:** Multivariant logistic regression analysis.

Multivariant Logistic Regression Analysis			
Analysis 1	variable	LL	*p* = 0.070
	covariable	SAPS3	*p* = 0.059

Abbreviations: LL = Last Lactate; SAPS3 = SAPS-3 score.

## Data Availability

The data that support the findings of this study are not openly available due to reasons of sensitivity, but are available from the corresponding author upon reasonable request.

## Supplementary Materials

The following supporting information can be downloaded at: https://www.mdpi.com/article/10.3390/jcm13071932/s1, Table S1: Diagnostic Characteristics of Lactate Variables & SAPS; Table S2: DeLong Test of significant Lactate Variables (*n* = 64); Table S3: Exploratory DeLong Test including FL (*n* = 64) and SAPS3 (*n* = 55).
