# Supplementary material for: Lactate as a Predictor of 30-Day Mortality in Cardiogenic Shock
Source: J Clin Med. 2024 Mar 27;13(7):1932. doi: 10.3390/jcm13071932 (PMC11012851; doi:10.3390/jcm13071932)
Supplement: Supplementary file 1 [file jcm-13-01932-s001.zip › jcm-2922230-supplementary.pdf]

Table S1: Diagnostic Characteristics of Lactate Variables & SAPS

Cut off values	n=	AUROC (p)*	Sensitivity	Specificity	PPV	NPV
FL [mmol/L]	64	0.567 (0.388)				
>2	50		87.0 %	26.8 %	40.0 %	78.6 %
>5	20		39.1 %	73.2 %	45.0 %	68.2 %
>5.5 (YI)	18		39.1 %	78.1 %	50.0 %	69.6 %
>6.7	10		26.1 %	90.2 %	60.0 %	68.5 %
>10	5		8.7 %	92.7 %	40.0 %	64.4 %
>15	1		4.3 %	100.0 %	100.0 %	65.1 %
LL [mmol/L]	64	0.743 (< 0.001)*				
>2	31		73.9 %	65.9%	54.8 %	81.8 %
>2,4	23		60.9 %	78.1 %	60.9 %	78.0 %
>3.1 (YI)	16		52.2 %	90.2 %	75.0 %	77.1 %
>5	9		26.1 %	92.7 %	66.7 %	69.1 %
>10	4		13.0 %	97.6 %	75.0 %	66.7%
PL [mmol/L]	64	0.655 (0.027)*				
>2	55		95,7 %	19,5 %	40,0 %	88,9 %
>5 (YI)	27		60,9 %	68,3 %	51,9 %	75,7 %
>10	11		26,1 %	87,8 %	54,5 %	67,9 %
>15	2		4,3 %	97,6 %	50,0 %	64,5 %
>20	1		4,3 %	100,0 %	100,0 %	65,1 %
LC [%]	64	0.672 (0.018)*				
<80	59		95.7 %	9.8 %	37.3 %	80.0 %
<65	52		91.3 %	24.4 %	40.4 %	83.3 %
<50	42		78.3 %	41.5 %	42.9%	77.3 %
<35	30		60.9%	61.0 %	46.7 %	73.5 %
<20	20		52.2 %	80.5 %	60.0 %	75.0 %
≤4.55 ≙ <10 (YI)	16		47.8 %	87.8 %	68.7 %	75.0 %
<0	14		39.1 %	87.8 %	64.3 %	72.0 %
SAPS3 [Points]	55	0.681 (0.023)*				
>60	39		76.2 %	32.4 %	41.0 %	68.7 %
>77	23		61.9 %	70.6 %	56.5 %	75.0 %
>79 (YI)	20		61.9 %	79.4 %	41.0 %	68.7 %
>85	12		42.9 %	91.2 %	75.0 %	72.1 %
>97	2		9.5%	100.0 %	100.0 %	64.2 %

* … Significant result in the AUROC with $p < 0.05$; Abbrevations: AUROC = Area Under the Receiver Operating Characteristics Curve with as the asymptotic significance; LL= Last Lactate; LC= Lactate Clearance; PL= Peak Lactate; FL = First Lactate; YI = Youden's Index; PPV = Positive Predictive Value; NPV = Negative Predictive Value;

Table S2: DeLong Test of Significant Lactate Variables (n = 64)

DeLong Test	p	ΔAUC in ROC	Standard error	95%-CI		z statistic
**LL vs. PL**	0.140	0.088	0.059	-0.029	0.021	1.475
**LL vs. LC**	0.333	0.071	0.073	-0.073	0.215	0.968
**PL vs. LC**	0.877	0.016	0.106	-0.192	0.225	0.155

*= significant difference with $p < 0.0167$; Abbrevations: LL= Last Lactate; LC= Lactate Clearance; PL= Peak Lactate; FL = First Lactate; AUC= Area under the curve; ROC= Receiver operator characteristic; CI= Confidence interval;

Table S3: Exploratory DeLong Test Including FL (n = 64) and SAPS3 (n = 55)

DeLong Test	p	ΔAUC in ROC	Standard error	95%-CI		z statistic
**FL vs. LL**	0.019*	0.176	0.075	0.029	0.323	2.349
**FL vs. PL**	0.066	0.089	0.048	-0.006	0.183	1.838
**FL vs. LC**	0.417	0.105	0.129	-0.148	0.358	0.812
**SAPS vs. FL**	0.372	0.090	0.101	-0.108	0.288	0.894
**SAPS vs. LL**	0.321	0.102	0.102	-0.099	0.302	0.993
**SAPS vs. PL**	0.903	0.013	0.104	-0.191	0.216	0.122
**SAPS vs. LC**	0.927	0.011	0.115	-0.214	0.235	0.092

*= significant at $p < 0.05$; Abbrevations: LL= Last Lactate; LC= Lactate Clearance; PL= Peak Lactate; FL = First Lactate; AUC= Area under the curve; ROC= Receiver operator characteristic; CI= Confidence interval;
